# Allograft Reconstruction for Sarcomas of the Tibia

**DOI:** 10.2174/1874325001711010189

**Published:** 2017-03-22

**Authors:** Vincent Y. Ng, Philip Louie, Stephanie Punt, Ernest U. Conrad

**Affiliations:** 1 Department of Orthopaedics, University of Maryland Medical Center, 110 S. Paca St, 6^th^ Floor, Baltimore, Maryland 21201, United States; 2 Department of Orthopaedics, Rush University, Chicago, United States; 3 Department of Orthopedics and Sports Medicine, University of Washington, 1959 NE Pacific Street, Seattle, Washington 98195, United States

**Keywords:** Tibia, Allograft, Reconstruction, Complication, Sarcoma, Function

## Abstract

**Background:**

Allograft reconstruction of oncologic resections involving the tibia can have unpredictable results. Prior studies have reported a high rate of complications and a long recovery period involving prolonged bracing, repeated procedures and extended periods of antibiotics.

**Methods:**

The case details of 30 tibial allografts (12 adults, 18 children; 20 intercalary, 7 hemicortical, 3 other) were reviewed retrospectively. Based on factors including function, pain, healing and infection, clinical outcomes were stratified into three categories: excellent, moderate, and poor.

**Results:**

The overall survival rate of the allografts was 66% at a mean follow-up of 42 mos (adults) and 63 mos (children). Healing for metaphyseal junctions was successful in 73% at a mean of 44 weeks and for diaphyseal junctions, 64% at 41 weeks. Intercalary allografts in adults (4 of 20) all became infected and none had excellent results. All hemicortical allografts were performed in adults and 6 of 7 had excellent results. Distal intercalary allografts in children (6 of 20) had either excellent or moderate results with no infections, but had 3 nonunions and 2 fractures. Proximal intercalary allografts in children (8 of 20) had 2 excellent results, but had 6 infections requiring a cement spacer. Five of the six spacers were ultimately revised to another allograft or an arthroplasty.

**Conclusion:**

For tibial allograft reconstruction, surgeons and patients should prepare for a prolonged treatment course that may include multiple complications and surgeries. Excellent or moderate results can be achieved eventually in most, but amputation may be necessary in 15-20% of cases.

## INTRODUCTION

Improved oncologic outcomes with current treatment modalities for sarcoma underscore the need for durable and functional reconstructive surgical options [1]. Malignancies involving the tibia present a unique challenge because of limited soft tissue coverage and a propensity for delayed bone healing [2]. Depending on the site of disease, reconstructive options include allograft, extracorporeal irradiated autograft, amputation, endoprosthesis, distraction osteogenesis, vascularized autograft, and rotationplasty. Despite unpredictable healing, allograft has traditionally been one of the treatments of choice for limb salvage, particularly for diaphyseal lesions. When successful, allograft reconstruction can ultimately provide good to excellent long-term outcomes [3, 4].

Allograft reconstruction of the tibia is often fraught with complications, particularly in the first few years after surgery [5]. The triad of infection, nonunion and fracture accounts for the vast majority of adverse events [6]. Patients frequently must endure prolonged bracing and activity limitation, repeated operative procedures, and extended courses of antibiotics. These endeavors require great patience and determination not only of the patient and surgeon, but of the family and entire treatment team as well.

The purpose of this study was to examine the treatment courses and long-term outcomes of tibial allograft reconstruction in children and adults with sarcoma. Being able to characterize patterns of success and failure allows surgeons to effectively counsel patients and manage expectations prior to surgery.

## MATERIALS AND METHODS

Between 1997 and 2013, 30 tibial allografts were performed in 12 adults and 18 children (≤18 years old) by a single surgeon (EUC). All data was collected retrospectively in accordance to IRB protocol (Table **1**). The mean length of follow-up was 55.0 months. Recorded case details included neoadjuvant or adjuvant treatment, margin status (microscopically positive or negative), type of allograft, length of allograft, method of fixation, postoperative complications, time to union, reoperations, use of gastrocnemius flap, and status of allograft. Types of host-allograft junctions included those located in the proximal or distal metaphysis and in the diaphysis. Types of grafts included intercalary (proximal, distal or diaphyseal), osteoarticular, and hemicortical. Union was defined as disappearance of the allograft-host junction or bridging bone across three cortices on AP and lateral radiographs. The entirety of the treatment course was considered in categorizing overall outcome pertaining to the allograft (Table **2**).

All allografts were age-, size- and side-matched to the recipient. They were fresh frozen, sterile-packaged, non-irradiated, and liquid nitrogen stored. They were thawed in warm saline solution immediately before usage. Physeal or articular-sparing intercalary allografts were attempted in all cases in which there was at least 1 cm of uninvolved bone between the proposed osteotomy and the physis or subchondral bone. A favorable response to chemotherapy, a major determinant for risk of local recurrence, assessed by preoperative magnetic resonance imaging or positron emission tomography was also required for intercalary resection. Future limb length discrepancy was minimized by utilizing allografts 0.5 to 1 cm longer than the osseous resection. All patients were non-weightbearing and protected in a brace or walking boot for at least 3 months postoperatively. Activty and weightbearing were progressed based on the radiologic appearance of the allograft-host junctions and clinical symptoms.

## RESULTS

The overall survival rate of the original allograft was 66% (20/30). Of the 10 which failed, 4 resulted in an amputation. Seven of the 10 received cement spacers. One required an amputation for local recurrence, 5 were revised to a second allograft, and 1 received a total knee arthroplasty. The mean time to initial revision was 70 weeks and second revisions at an additional 64 weeks. Amputations performed for allograft failure were performed at a mean of 140 weeks. There were two local recurrences (7%). All patients except one had histologically negative osseous margins and all had either negative or microscopically positive soft tissue margins. Re-resection of margins unless they involved critical neurovascular structures were typically performed to achieve negative final margins.

There were 7 hemicortical resections, all of which were in adults (Table **3**). Five patients (2 adamantinoma, 2 Ewing sarcoma, 1 high grade soft tissue sarcoma [HG STS]) had excellent allograft results. The two patients with adamantinoma received no adjuvant treatment, the two patients with Ewing sarcoma received chemotherapy, and the one patient with HG STS received surgery only. A different patient with HG STS who received chemoradiation also had an excellent clinical outcome, but required a prolonged course of intravenous antibiotics.

There were 20 intercalary resections (4 adults, 16 children). None of the adults had an excellent outcome and all acquired deep infections. In children, two patients had diaphyseal allografts (1 excellent, 1 moderate outcome). Six patients had distal intercalary allografts (4 excellent, 2 moderate) and 8 had proximal intercalary allografts (2 excellent, 6 poor). None of the distal intercalary grafts became infected, but three had nonunions and two had fractures. Of the six poor outcomes for the proximal intercalary grafts, all were for infection and were explanted for a cement spacer. Nevertheless, five of the six were ultimately able to receive either a new allograft or a total knee arthroplasty after numerous surgeries. The sixth underwent an amputation for local recurrence.

For metaphyseal junctions, 73% healed at a mean of 44 weeks (S.D., 24 wks) after the initial allograft reconstruction and if necessary, 30 weeks (13 wks) after a revision surgery for nonunion. For diaphyseal junctions, 64% healed at a mean of 41 weeks (14 wks) after the initial allograft reconstruction and if necessary, 52 weeks (8 wks) after a revision surgery for nonunion. There were no statistically significant differences in healing for location, gender or age at surgery.

Four medial gastrocnemius flaps were performed. One was for a proximal tibial hemicortical allograft after neoadjuvant radiation and resection of a HG STS. This patient had an excellent outcome, but required prolonged antibiotics for cellulitis. Two were performed in conjunction with surgical debridement for infection in proximal intercalary allografts that were explanted for a cement spacer. Both cases were salvaged with an allograft or subsequent arthroplasty. One gastrocnemius flap was performed for a diaphyseal intercalary allograft with wound healing delay. This patient required an amputation for distal diaphyseal wound failure and progression of disease.

Of the 7 skeletally immature children with moderate or excellent results who received intercalary allografts, 3 were physeal-sparing. Two of three of these patients demonstrated subsequent longitudinal growth from the physis of interest.

## DISCUSSION

This study emphasizes the unpredictable nature of tibial allograft reconstruction, but also sheds light on several trends based on age of the patient and the type of the allograft. In adults, hemicortical allografts have limited complications and positive results. With other types of allografts, adult patients experience many complications and achieve, at best, moderate results. In children, proximal intercalary allografts have certain advantages relative to megaprostheses, but are highly susceptible to infection. Excellent results without complications are possible in the minority cases. For those that require cement spacers and prolonged antibiotics, eradication of infection and reimplantation of an allograft or arthroplasty are possible. Distal intercalary allografts are less prone to infection, but have high rates of nonunion and fracture. Diaphyseal grafts have longer time to union and higher nonunion rates. Limitations of this study include a relatively heterogeneous sample and small sample size due to the rare nature of this condition and caution must be taken when interpreting results.

Allografts are affected early by a triad of complications: infection, nonunion, and fracture. If the patient, surgeon, and allograft can endure the first 3 to 4 years after allograft reconstruction, the construct is generally very durable and prone to few complications. Particularly for allograft reconstruction of tibial lesions, patients and their families should be well-apprised of the necessity for secondary procedures in nearly half of all cases [3, 7], the presence of at least one complication in the majority of patients [8], the prolonged time to union and limited weightbearing of at least 9 months [6, 9, 10], and the 5-15% risk of amputation [8, 9].

Reported rates of nonunion range from 6% to 63% [5, 7, 11, 12], with a higher rate at diaphyseal than metaphyseal junctions [7, 9]. Treatment with autograft and revision of hardware appears to be effective, but may require multiple attempts in at least 30% -50% [9, 12, 13]. Nonunion in itself typically does not portend ultimate allograft failure unless the allograft becomes infected [7, 12]. Prolonged time to union and multiple revision procedures may be necessary [9].

Vascularized fibula allograft supplementation has been described to possibly decrease the risk of nonunion. Theoretically, the main structural allograft protects the vascularized fibula initially from fracture. As creeping substitution and resorption takes place, the fibula hypertrophies and compensates for the weakened structural graft until full incorporation takes place. Nevertheless, fracture, infection, prolonged operative time, and donor site morbidity are still frequent issues [6, 13, 14].

Compression plating and absolute stability are reported to have lower nonunion rates than intramedullary nails and relative stability [13]. Gaps greater than 2 mm are associated with nonunion [12]. Plate fixation has higher rates of allograft fracture than intramedullary fixation. This is likely related to more screw holes and stress risers. Spanning the entire graft with the plate may reduce the risk of fracture [7, 11, 15], and some authors even advocate double plate fixation for the junction site or intramedullary cementation [8]. Reported rates of allograft fracture range from 10% to 20% [11, 12, 16]. Most fractures occur between 5 months and 3 years, corresponding to the period of partial graft resorption before deposition of vascularized bone [5]. Although allograft-allograft healing is possible with internal fixation and autografting [13, 15, 16], complete and displaced fractures frequently result in allograft failure and revision [12, 15].

The reported incidence of infection for lower extremity allografts range from 6-30% [5, 7, 9, 12, 17]. Tibial allografts have a very limited soft tissue envelope and different measures such as primary medial gastrocnemius flaps [3, 16], extended courses of prophylactic antibiotics, and antibiotic soaks prior to allograft implantation may be helpful [11]. Fresh frozen, sterile packaged, non-irradiated, liquid-nitrogen stored allografts from a reputable tissue bank should be used. A two stage exchange with a cement spacer and intravenous antibiotics appears effective in eradicating the infection and allowing reconstruction with a new allograft or arthroplasty for good to excellent functional outcome [3, 15, 18].

Osteoarticular allografts have a 40% to 60% failure rate at 5 years and universal eventual joint degeneration [5, 19]. Alloprosthetic composites have poor results, high complication rates, and are generally not recommended for the knee. Both options have largely been replaced by megaprostheses in the United States. In children, preserving the articular cartilage, the distal femoral physis and possibly the proximal tibial physis with an intercalary allograft have obvious advantages with respect to minimizing limb-length discrepancy. Modern MRI techniques are highly accurate at determining the extent of tumor involvement of the epiphysis and assessing the feasibility of intercalary resection [18]. Close but negative bone margins can be achieved in the majority of cases, but positive margins should be re-resected to achieve final negative margins.

Reconstructive options for the distal tibia are more limited as there are no widely available prosthetic implants and allografts are often the only choice aside from amputation. The possibility for excellent outcomes in children justifies an attempt at intercalary reconstruction despite the specter of multiple procedures and protracted treatment courses. Amputation should be discussed as a reasonable option in patients with challenging distal tibial reconstructions because of the relatively high function of below-knee amputations. In adults, the failure of intercalary allografts is higher and the need to reduce future limb-length discrepancy is not relevant. Megaprosthetic options should be strongly considered in adults for proximal tibial lesions. For soft tissue sarcomas with periosteal involvement and some limited cortically-based primary bone sarcomas, hemicortical allografts generally fare well and are viable solutions. Chemotherapy and radiation therapy, when appropriate, can improve survival when combined with surgical resection.

Because of the limited reconstructive options for the tibia, allografts will always be animportant yet challenging tool in the orthopaedic oncologist’s armamentarium. Complications and failure in a substantial portion of cases are difficult to avoid. Patients, families, and surgeons alike should be aware of the potentially long haul ahead whenever embarking down this road.

## Figures and Tables

**Table 1:** Demographics of 30 patients with tibial allografts.

	**Adults**	**Children**
**Number of patients**	12	18
**Mean Age at Initial Surgery (yrs, range)**	48 (27 – 76)	13 (7 – 18)
**Mean Length of Follow Up (mos, range)**	43 (4 – 121)	63 (19 – 175)
**Diagnoses (n)**	Adamantinoma (2) HG STS with osseous involvement (3) Osteosarcoma (2) Ewing sarcoma (2) IG STS (2)	Osteosarcoma (8) Ewing sarcoma (7) Adamantinoma (2) Primitive mesenchymal sarcoma (1)
**Type of allograft (n)**	Hemicortical (7) Intercalary (4 diaphyseal) Distal osteoarticular (1)	Intercalary (8 proximal, 6 distal, 2 diaphyseal) Distal osteoarticular (1) Hemiresection osteoarticular (1)
**Mean Length of allograft (cm)**	Hemicortical (9.1) Intercalary (9.5 diaphyseal) Distal osteoarticular (10)	Intercalary (8.4 proximal, 12.5 distal, 17.5 diaphyseal) Distal osteoarticular (10) Hemiresection osteoarticular (8)
**Mode of fixation**	11 plate/screws, 1 IMN	17 plate/screws, 1 IMN

HG STS – high grade soft tissue sarcoma IG STS – intermediate grade soft tissue sarcoma IMN – intramedullary nail

**Table 2:** Overall outcome criteria of allograft reconstruction^A^.

**Category**	**Criteria**
**Excellent**	Allograft union AND *all* of the following: Minimal to no pain requiring no pain medications Normal to near-normal ambulation No usage of suppressive antibiotics No reoperations for nonunion, fracture or infection^B^
**Moderate**	Allograft union AND *any* of the following: Moderate pain requiring non-narcotic pain medication Significant limp or requires walking aid or brace Significant malalignment or articular degeneration Chronic suppressive oral antibiotics
**Poor**	*Any* of the following: Chronic nonunion despite reoperations Loss of original allograft due to nonunion, fracture or infection Significant pain requiring chronic narcotic usage Inability to ambulate outside of the house

^A^at latest follow-up ^B^excludes reoperations not directly related to allograft reconstruction such as epiphysiodesis, removal of symptomatic, loose, or physeal-crossing hardware, or resection of local recurrence.

**Table 3:** Allograft outcome based on type.

**Type of Allograft**	**Excellent**	Excellent*	**Moderate**	**Poor**
Hemicortical	5	1	0	1
Intercalary	5	2	5	8
Proximal	2	0	1	6
Diaphyseal	1	0	1	2
Distal	2	2	3	
Distal Osteoarticular	0	0	0	2
Hemiresection Osteoarticular	0	0	1	0

*Excellent outcome, but required either operative/nonoperative treatment for nonunion, fracture or infection, or a prolonged course (>1 wk) of intravenous antibiotics

## ABBREVIATION

HG STS: = High grade soft tissue sarcoma
